# Research on the innovative application of Shen Embroidery cultural heritage based on convolutional neural network

**DOI:** 10.1038/s41598-024-60121-7

**Published:** 2024-04-26

**Authors:** Jiajun Zhu, Changyong Zhu

**Affiliations:** School of Computer and Information Engineering, Nantong Institute of Technology, Nantong, China

**Keywords:** Shen Embroidery, Convolutional neural network, MobileNet V1, Transfer learning, Innovative research, Computer science, Information technology

## Abstract

In order to protect intangible cultural heritage and promote outstanding cultural works, this article introduces innovative research on Shen Embroidery using convolutional neural networks. The dataset of Shen Embroidery was preprocessed to augment the data required for experimentation. Moreover, the approach of transfer learning was introduced to fine-tune the recognition network. Specifically, Spatial Pyramid Pooling (SPP) is employed by replacing the avg pool in the MobileNet V1 network, achieving the fusion of local and global features. The experimental results showed that the improved MobileNet V1 achieved a recognition accuracy of 98.45%, which was 2.3% higher than the baseline MobileNet V1 network. The experiments demonstrated that the improved convolutional neural network can efficiently recognize Shen Embroidery and provide technical support for the intelligent development of intangible cultural heritage.

## Introduction

Shen Embroidery, as a product of the integration of excellent traditional Chinese craftsmanship and the development of modern textile industry. Originated in "Nantong", the birthplace of modern textile industry along the coast of China. Its founder is the renowned embroidery artist Shen Shou^1^. However, with the development of society, the impact of modern industry on traditional handicrafts has posed a significant threat to the preservation of traditional Shen Embroidery techniques. Therefore, it has become extremely urgent to employ various methods to identify, protect, and pass on Shen Embroidery as intangible cultural heritage from generation to generation.

In recent times, the protection and inheritance of Shen Embroidery in China have typically relied on manual methods to identify, select, and classify. These tasks are often laborious and require a significant amount of human and financial resources. However, with the development of computer processing methods and the emergence of computer vision technology^2^. Computer technology can automatically learn and understand various forms of visual data, such as photos and videos. Among these computer vision techniques, image classification involves categorizing images into different classes based on their features and visual characteristics^3^. By leveraging computer vision technology, Shen Embroidery can be effectively and automatically learned and distinguished to better identify different types of embroidery. Ming et al.^4^ proposed a new clustering algorithm that combines the SC clustering algorithm with automatic classification of cloud pattern images and integrates SC characteristics with ANP-MEAP to achieve automatic classification of cloud pattern images. Zhe et al.^5^ introduced a Cantonese embroidery classification method based on the K-means clustering algorithm, which identifies Cantonese embroidery by evaluating the saliency of color features. Tajeripour et al.^6^ proposed an image retrieval framework based on texture analysis technology, which can retrieve digital images from a vast database and identify them based on features such as color and shape. While traditional machine vision techniques have achieved a series of results, they often require manual annotation of key features in images. Since different annotators may have different interpretations and descriptions of images, the resulting keyword annotations may also vary. As a result, the accuracy and efficiency of this classification method are greatly influenced by the annotators, leading to poor accuracy and unsatisfactory results.

With the advancement of technology and the rise of artificial intelligence, convolutional neural network (CNN) have further developed^7^. Researchers have utilized CNN for image recognition^8^, object detection^9^, and semantic segmentation^10^. These techniques are applied primarily in various aspects of life^11^, production^12^, and healthcare^13^. Currently, there are various architectures and variants of CNNs that enhance deep learning performance. These variants, such as VGGNet^14^, ResNet^15^, and Xception^16^, exhibit significant improvements in image classification accuracy and have achieved great success in many image classification tasks. In the realm of intangible cultural heritage preservation, researchers have begun integrating deep learning techniques into cultural inheritance and protection. Chen et al.^17^ proposed a Cantonese opera Genre Classification Networks (CoGCNet) model for classifying Cantonese opera singing types, combining a bi-layer long short-term memory network (LSTM) with a conventional neural network (CNN) to enhance the contextual relevance between signals. This approach achieved intelligent classification management of Cantonese opera data with an accuracy of 95.69%. Zhou et al.^18^ applied deep learning and transfer learning techniques to embroidery images, fine-tuning the Xception model for classification and recognition of embroidery images, addressing the problem of insufficient embroidery data collection in traditional Chinese embroidery. Wang et al.^19^ introduced an innovative design method for willow pattern motifs, using ResNet to establish an image recognition model for Funan willow pattern, contributing to the sustainable development of willow craftsmanship culture. Experimental results showed that ResNet achieved the best recognition rate of 94.36% for the entire image dataset, with a recognition rate of 95.92% for modern patterns and 93.45% for traditional willow patterns. Yu et al.^20^ incorporated the AlexNet model into the application research of Nantong blue calico, utilizing data augmentation techniques to expand the collected texture samples. Based on the deep learning AlexNet model, the cultural connotations of texture patterns were analyzed, and the AlexNet model achieved high accuracy in classifying patterns of Nantong blue calico with a learning rate of 0.002.

In order to effectively address the issues of the limited quantity of Shen Embroidery, lack of datasets, and the labor-intensive process of manually selecting Shen Embroidery for classification and recognition. This study applies artificial intelligence technology to Shen Embroidery. Convolutional neural networks were used to recognize Shen Embroidery, assisting researchers in better studying Shen Embroidery and further protecting and inheriting intangible cultural heritage. The specific work in this study is as follows:

1. Enhancing and expanding the dataset through image processing techniques.
2. Experimenting and comparing five different image classification networks, followed by analysis and discussion.
3. Fine-tuning the classification network using transfer learning and conducting analysis and discussion.
4. Replacing "avg pool" with "Spatial Pyramid Pooling" (SPP) and analyzing the improved accuracy.

The application of these steps aimed to improve the classification and recognition of Shen Embroidery using artificial intelligence, providing valuable insights for the preservation and research of intangible cultural heritage.

## Materials and methods

### Dataset and experimental environment

**Figure 1:**
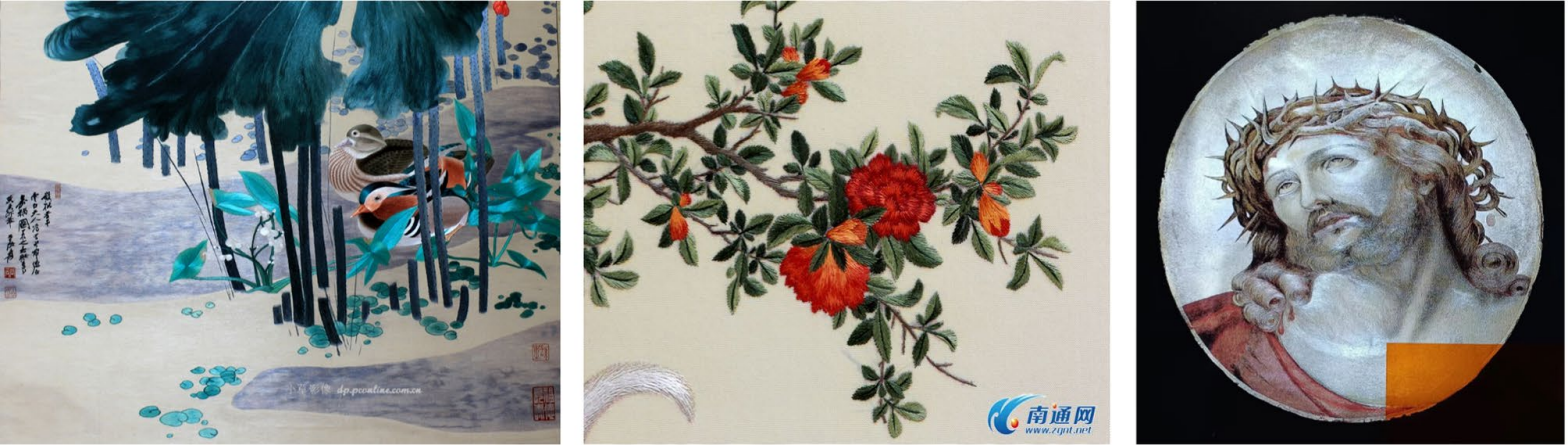
The image of dataset.

**Figure 2:**
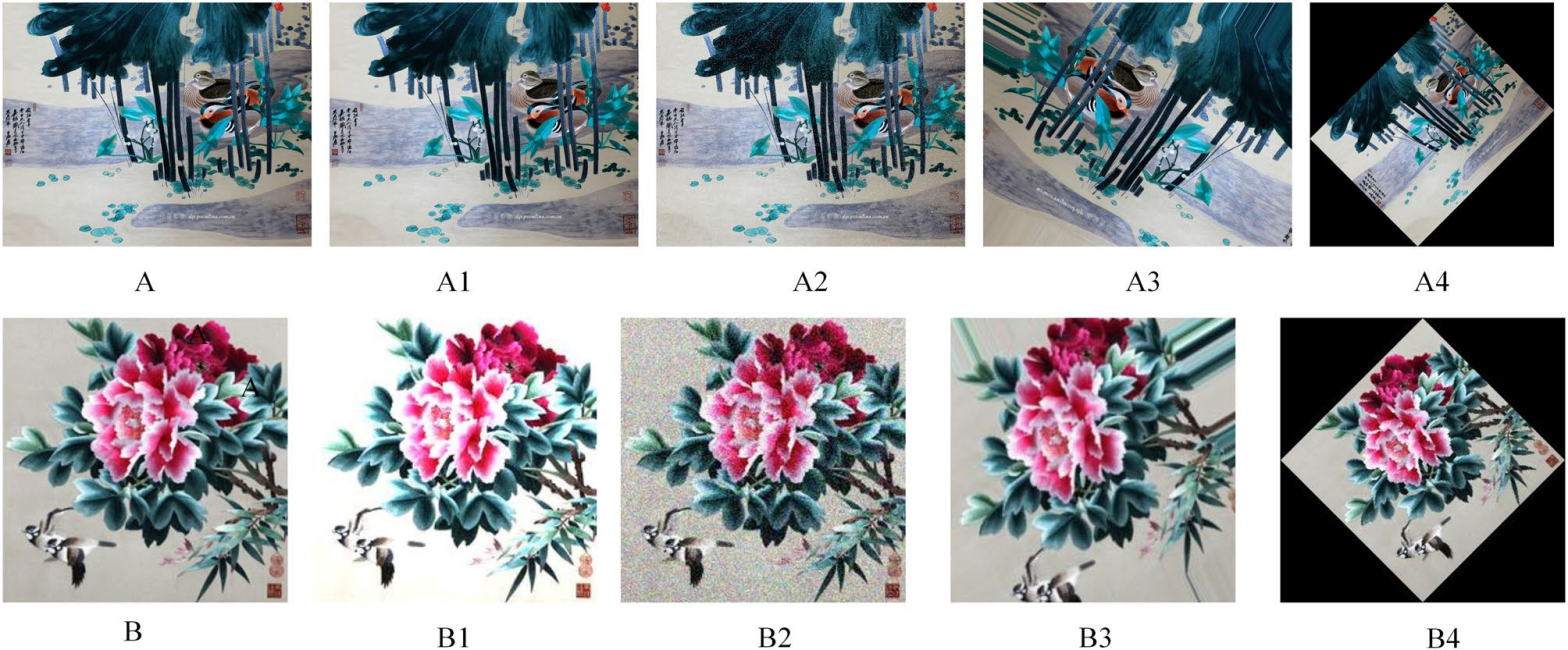
The image of data augmentation.

The majority of the experimental dataset in this study was obtained from the Shen Embroidery Museum located in Nantong, Jiangsu Province, China. Additionally, a portion of Shen Embroidery was collected through web scraping to augment the dataset. The dataset consists of a total of 1264 Shen Embroidery images with a uniform size of 224 × 224 pixels and in JPG format. Example images of the dataset were shown in Fig. 1. Before conducting the experiments, data augmentation techniques such as flipping, rotating, and color variation were applied to expand the dataset. The enhanced data images are depicted as shown in Fig. 2. On average, each image was augmented 15 times, resulting in a total of 18,960 augmented Shen Embroidery. The dataset was divided into a training set and a validation set in a 9:1 ratio for the experimental training process. The training set served as the input for network training. The training set is utilized as the network's training data. And the validation set serves as self-checking data for the network's learning process. Furthermore, a separate test set consisting of 100 images containing Shen Embroidery was collected to evaluate the performance of the trained model.

The experimental platform of this article was based on the Windows 10 operating system. Programming was done by using the Python language under Anaconda. The deep learning framework used was PyTorch. The main hardware configuration of the computer used for the experiments includes an 8 GB GPU GeForce GTX 1070Ti and an AMD Ryzen 5 1600X six-core processor. The number of epochs for network training is 200, the batch size is 4, the initial learning rate is 0.001, and the input image size is 224 × 224. The model was saved every 5 training iterations.

### MobileNet V1 network

MobileNet V1^21^ is a lightweight neural network designed for mobile devices, characterized by its small model size, low parameter count, and fast computation speed. In this paper, the lightweight neural network named MobileNet V1 was used for the recognition of Shen embroidery. The network structure of MobileNet V1 was shown in Table 1. MobileNet V1 consists of 28 layers and utilizes convolutional strides instead of pooling layers for downsampling. It also employs depthwise convolutions and batch normalization (BN) layers. The architecture of MobileNet V1 is a streamlined structure composed of standard convolutional layers (Conv Std), 13 depthwise convolutional layers (Conv dw), 13 pointwise convolutional layers (Conv pw), average pooling layer (avg Pool), and fully connected layer (FC). In the network, pooling operations are replaced by depthwise convolutions with a stride of 2, while the global average pooling layer is retained at the end of the network. Additionally, each convolutional layer is followed by a BN layer for batch normalization and ReLU activation function.

**Table 1:** The network structure of MobileNet V1.

Convolution type/step	Convolution kernel size	Input size
Conv/s2	3 × 3 × 3 × 32	224 × 224 × 3
Conv dw/s1	3 × 3 × 32 dw	112 × 112 × 32
Conv/s1	1 × 1 × 32 × 64	112 × 112 × 32
Conv dw/s2	3 × 3 × 64 dw	112 × 112 × 64
Conv/s1	1 × 1 × 64 × 128	56 × 56 × 64
Conv dw/s1	3 × 3 × 128 dw	56 × 56 × 128
Conv/s1	1 × 1 × 128 × 128	56 × 56 × 128
Conv dw/s2	3 × 3 × 128 dw	56 × 56 × 128
Conv/s1	1 × 1 × 128 × 256	28 × 28 × 128
Conv dw/s1	3 × 3 × 256 dw	28 × 28 × 256
Conv/s1	1 × 1 × 256 × 256	28 × 28 × 256
Conv dw/s2	3 × 3 × 256 dw	28 × 28 × 256
Conv/s1	1 × 1 × 256 × 512	14 × 14 × 256
Conv dw/s1	3 × 3 × 512 dw	14 × 14 × 512
5 × Conv/s1	1 × 1 × 512 × 512	14 × 14 × 512
Conv dw/s2	3 × 3 × 512 dw	14 × 14 × 512
Conv/s1	1 × 1 × 512 × 1024	7 × 7 × 512
Conv dw/s2	3 × 3 × 1024 dw	7 × 7 × 1024
Conv/s1	1 × 1 × 1024 × 1024	7 × 7 × 1024
Avg Pool/s1	Pool 7 × 7	7 × 7 × 1024
FC/s1	1024 × 1000	1 × 1 × 1024
Softmax/s1	Classifier	1 × 1 × 1000

### Transfer learning

**Figure 3:**
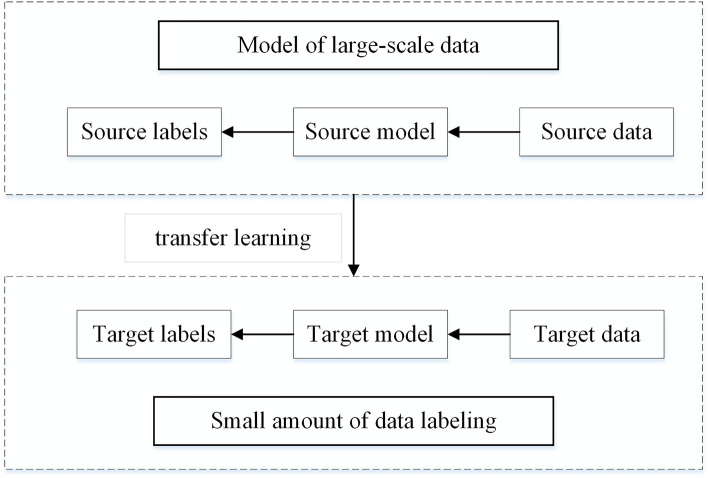
The concept of transfer learning.

Training the convolutional neural network (CNN) required a large dataset of images. However, in this study, the image of Shen Embroidery lacked obvious texture features such as shape and color. Therefore, high-fidelity images were required as the model dataset to better extract key texture features of Shen Embroidery. However, obtaining sufficient training data is difficult, and the cost of collecting labeled datasets is high^22^. CNN models such as AlexNet, ResNet, and Xception had been trained on large ImageNet datasets for image recognition. These models can recognize different tasks without the need for training from scratch. Pretrained models also aid in network generalization and accelerate convergence. Model fine-tuning refers to unfreezing the top layers of the pretrained model, allowing the learned features to be more relevant to the current task. Transferring the trained model to a new task and training it was known as transfer learning^23^. In this study, due to the limited Shen Embroidery, transfer learning was utilized to avoid excessive training parameters and reduce the risk of overfitting in the network model. Figure 3 illustrated the concept of transfer learning.

### Spatial Pyramid Pooling (SPP)

**Figure 4:**
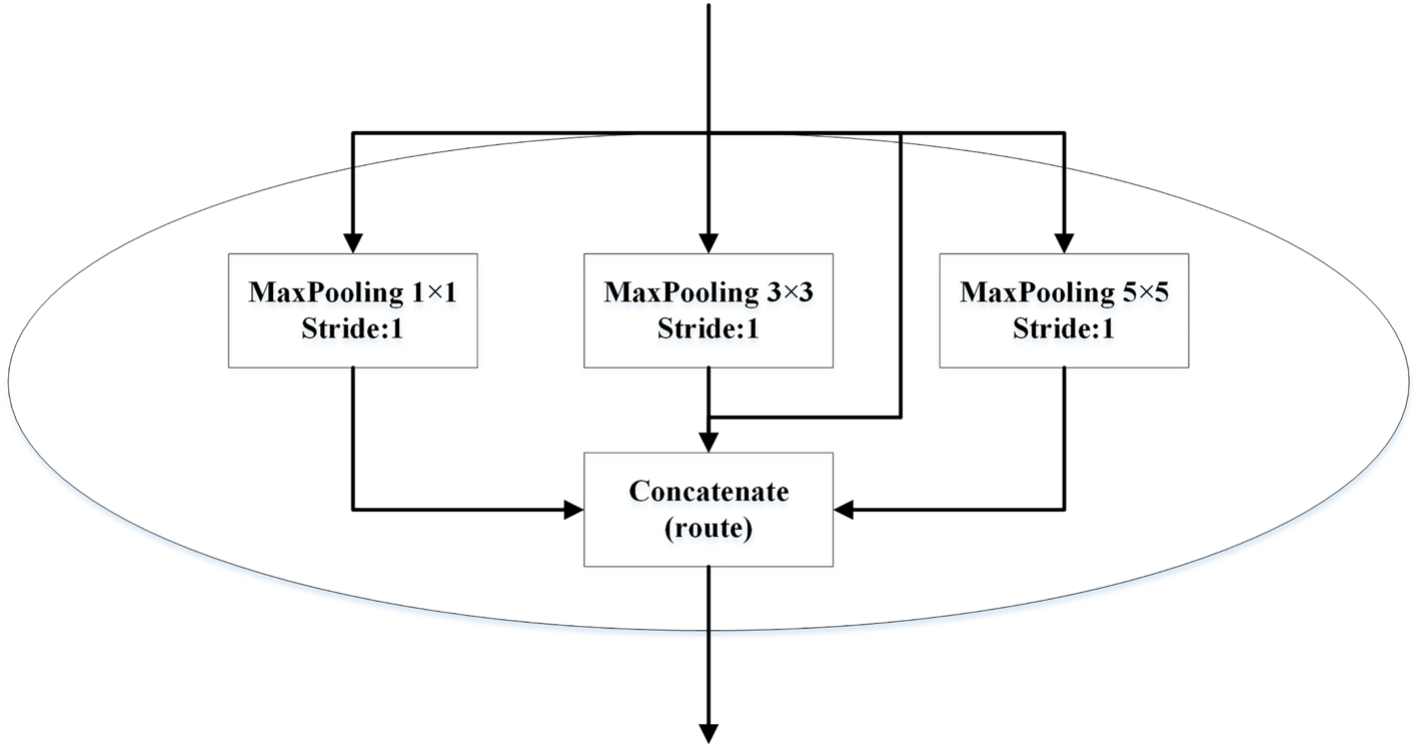
The module of SPP.

The Spatial Pyramid Pooling (SPP) module drawed inspiration from the spatial pyramid concept and enabled the fusion of local and global features. By integrating local and global features, the expressive power of the feature maps was enhanced, which was beneficial for improving detection accuracy in situations where there are significant variations in object sizes within the image. In the recognition network constructed in this study, the SPP module was incorporated before the feature output layer. The SPP module consisted of three components: max pooling with kernel sizes of 1 × 1, 3 × 3, and 5 × 5, and a concatenate operation. This configuration was illustrated in Fig. 4. The input to the SPP module was a 7 × 7 feature map obtained through convolution, and the output was the concatenation of the three parallel branches.

### Evaluation indicators

The evaluation metric for the model in this paper was accuracy (ACC), as shown in Eq. (1):

1 $$ACC = \frac{TP + TN}{{TP + FP + FN + TN}}$$

where, TP represents the number of correct identifications of "shenxiu," TN represents the number of correct identifications of "fei," FN represents the number of "shenxiu" mistakenly identified as "fei," and FP represents the number of "fei" mistakenly identified as "shenxiu."

## Results

### The training results of MobileNet V1

**Figure 5:**
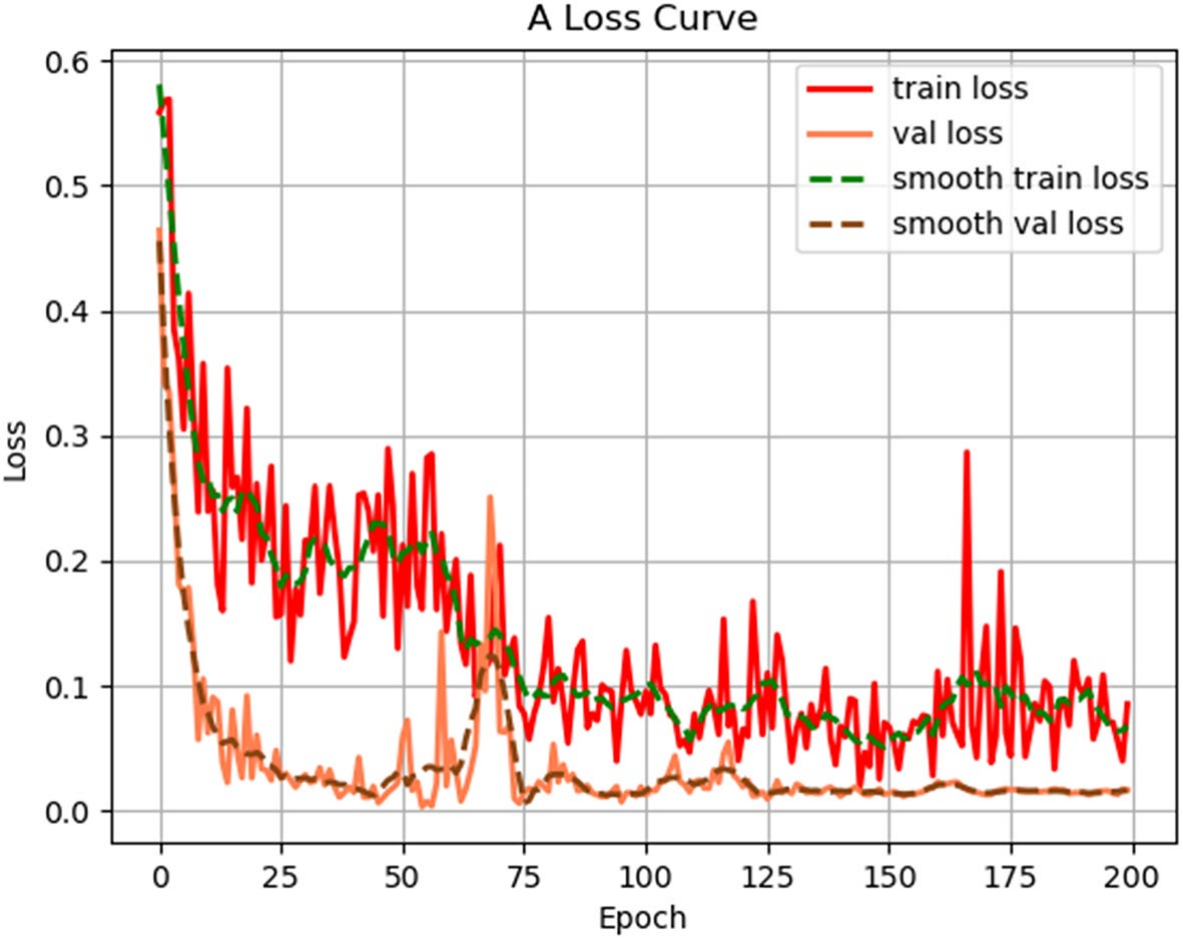
The training results of MobileNet V1.

For the recognition of "shenxiu" images, this study utilized the MobileNet V1 model for experimentation. During the experiment, a total of 200 epochs were trained, with a checkpoint saved every 5 epochs. Figure 5 illustrated the training results of the MobileNet V1 model, where the x-axis represented the training epochs and the y-axis represented the loss value. The convergence of the model can be determined by observing the change in the loss value, where a gradual stabilization of the loss indicates the convergence process. The red curve represents the train loss, while the green curve represents the validation loss. As the training epochs increase, the fluctuation in the loss curve gradually stabilizes. After around 100 epochs of training, the curve begins to level off, indicating that the model is approaching stability, with the loss value eventually stabilizing at around 0.1. However, it is worth noting that there is still significant fluctuation in the loss value during the process of approaching stability, as evident from the graph.

### The training results of improved MobileNet V1

**Figure 6:**
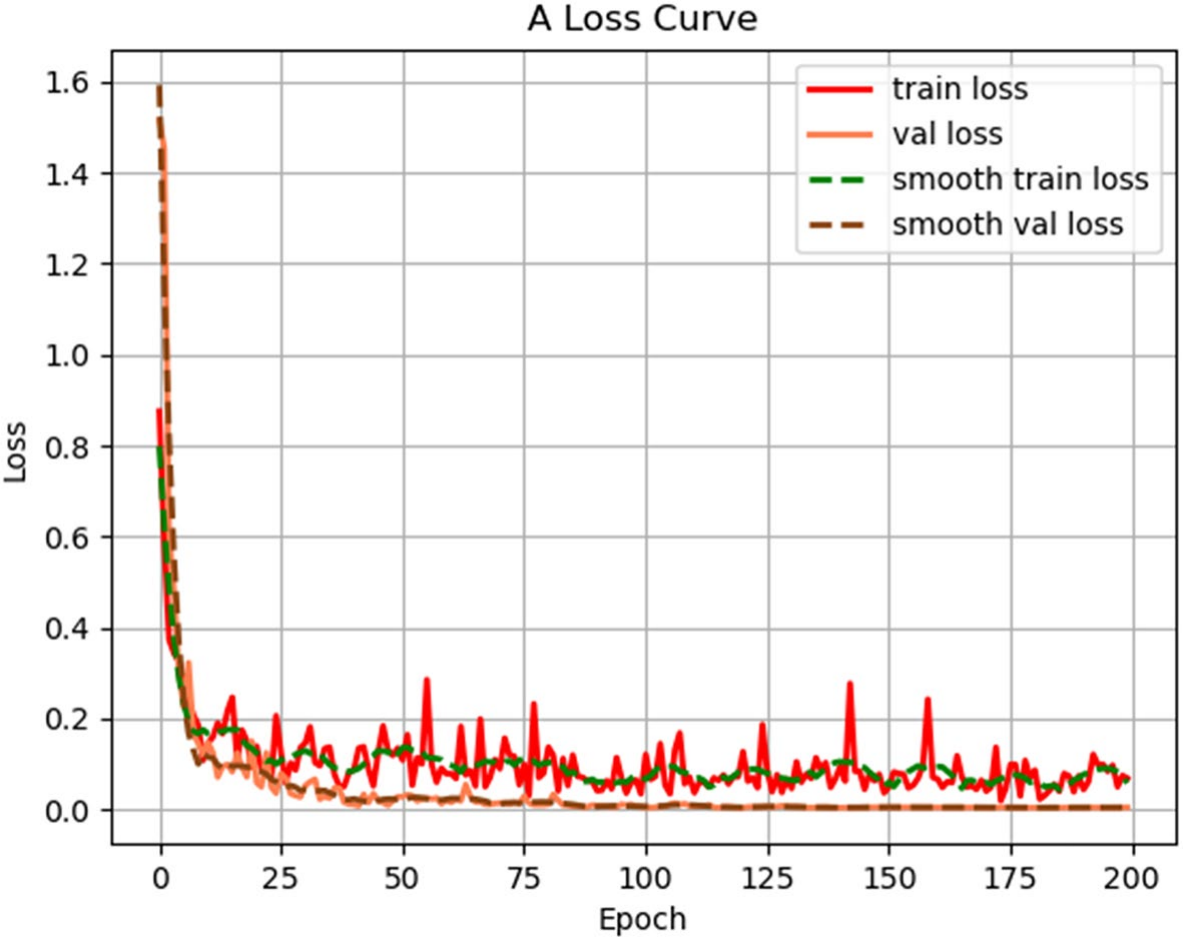
The training results of improved MobileNet V1.

For the recognition of "shenxiu" images, this study improved the MobileNet V1 model. The same dataset was fed into the modified MobileNet V1 network for training, with a total of 200 epochs and a checkpoint saved every 5 epochs. Figure 6 shown the training results of the improved MobileNet V1 model. The x-axis represents the training epochs, while the y-axis represents the loss value. The red curve represents the train loss, and the green curve represents the val loss. As the training epochs increase, the loss curves tend to stabilize. After 100 epochs, the curves reached a steady state, indicating a well-fitted model, with the loss value eventually stabilizing at around 0.12.

## Discussion

### Experimental comparison of different models

**Figure 7:**
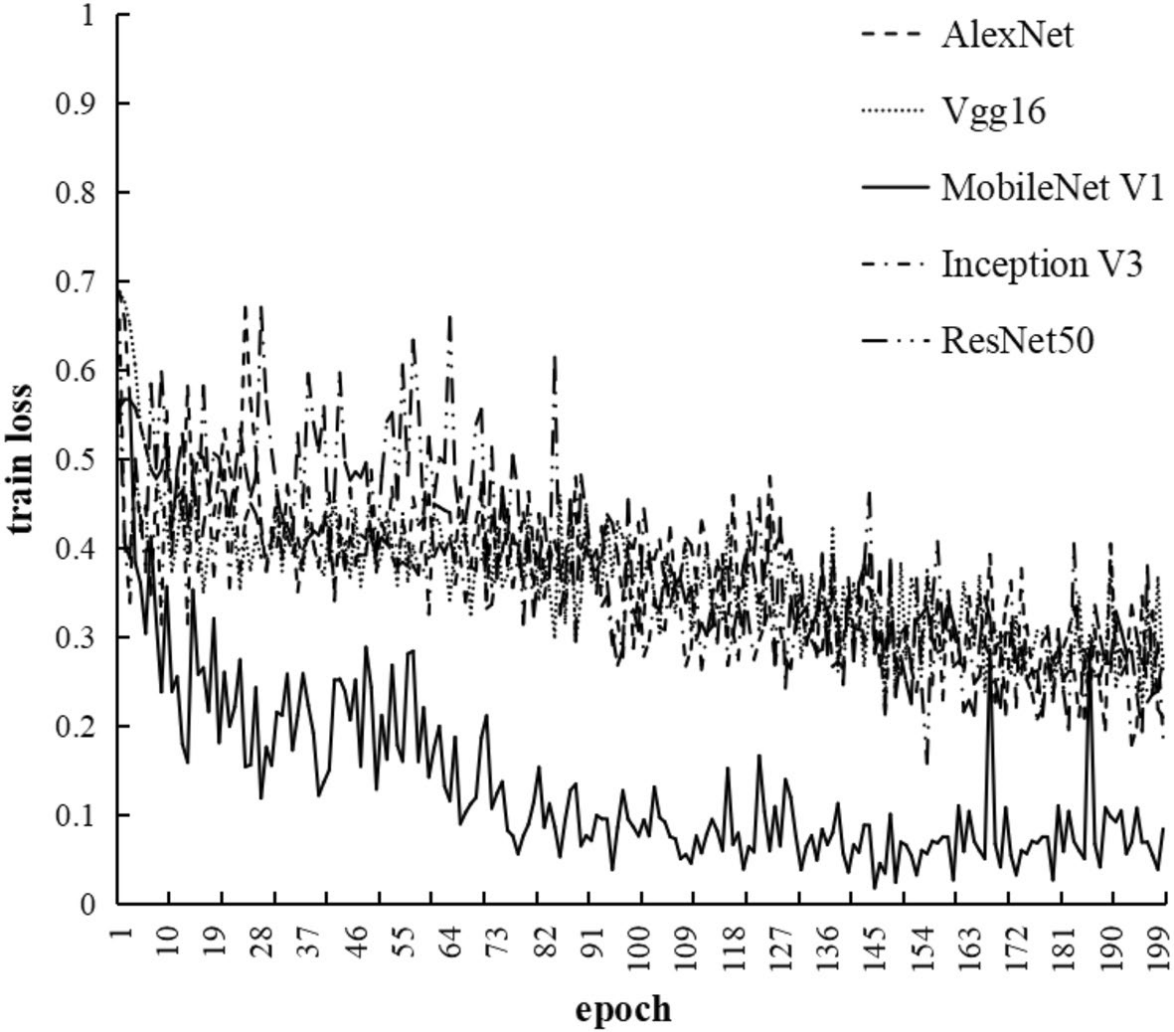
Comparison of train loss of different models.

**Figure 8:**
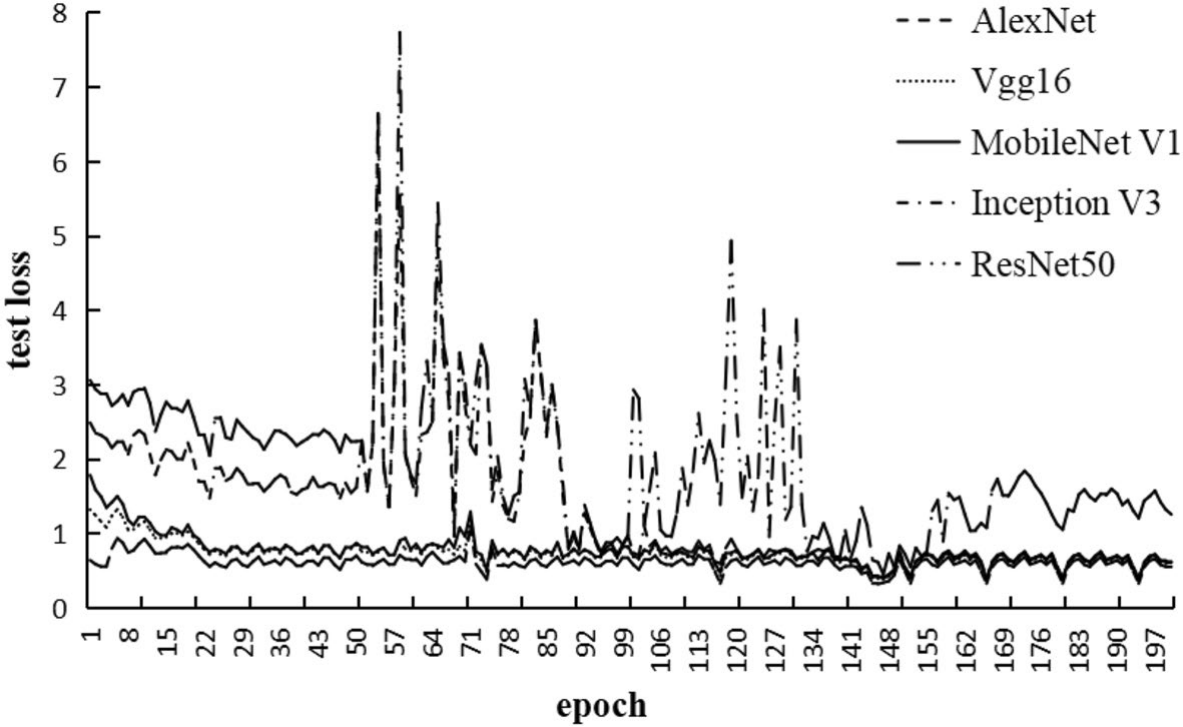
Comparison of test loss of different models.

This study conducted a horizontal comparison of several mainstream classification models, including AlexNet, VGG16, ResNet50, MobileNet V1, and InceptionV3. AlexNet model won the championship in the 2012 image classification competition. VGG16 model achieved first place in the 2014 ImageNet^24^ localization task. ResNet50 won the first place in the classification task of ILSVRC2015. MobileNet V1, proposed by Google in 2017, is a widely used lightweight network . InceptionV3^25^ was an upgraded version of GoogLeNet after achieving first place in ImageNet. Throughout the experiment, the training dataset, number of epochs, learning rate, batch size, and computer hardware were kept consistent. The loss values of the five classification models during the training process are shown in Fig. 7, with the x-axis representing the number of training iterations and the y-axis representing the loss values. It can be observed that the MobileNet V1 model converged the fastest, with the least fluctuation and the best performance during training. Figure 8 shown the loss variation of the five models on the validation set. From the graph, it can be seen that MobileNet V1 also performs well on the validation set. Table 2 represented the ACC of the five models during the training process, with MobileNet V1 achieving an ACC of 96.15%, higher than the other four models, indicating high recognition accuracy.In conclusion, the MobileNet V1 model can be selected as the recognition network for the Shen Embroidery in this study.

**Table 2:** Accuracy of different models.

Model	Accuracy (%)
AlexNet	88.23
VGG16	86.16
MobileNet V1	96.15
InceptionV3	92.38
ResNet50	93.35

**Figure 9:**
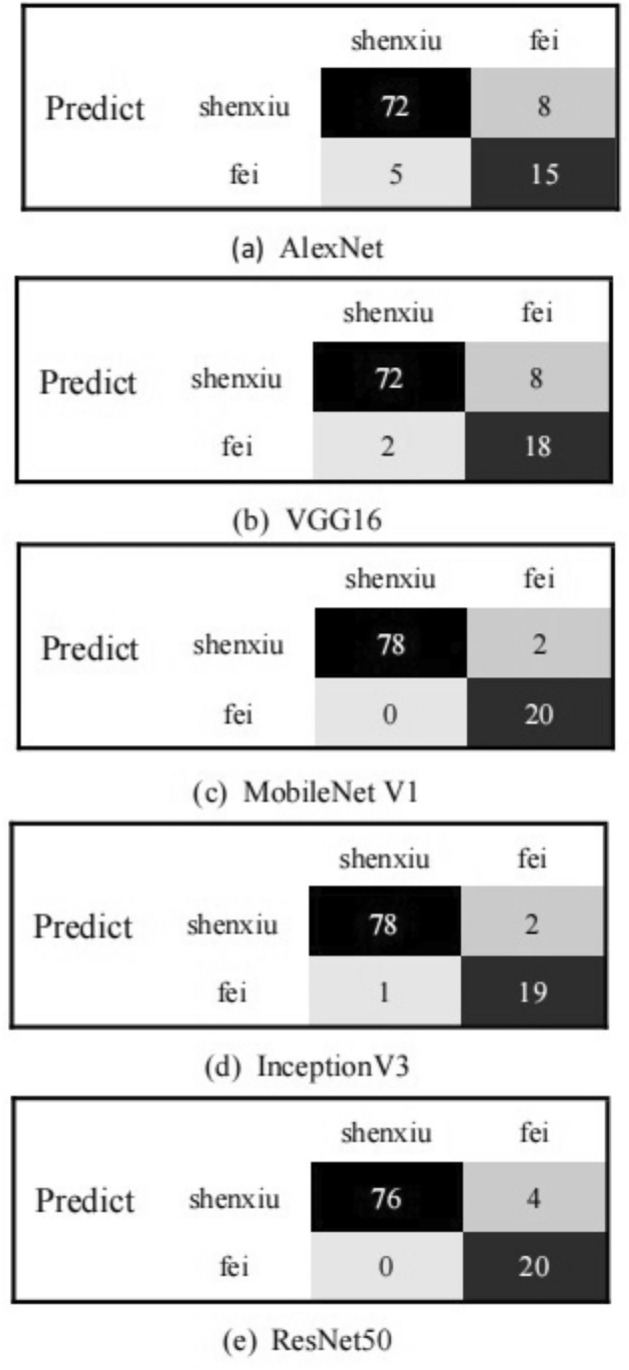
Confusing matrix.

After training and fitting, the five models were used to recognize the Shen Embroidery. A dataset of 100 images, including 80 images of Shen Embroidery and 20 images of non-Shen Embroidery, was used for testing. The expected correct recognition results should be "shenxiu" for the 80 images and "fei" for the 20 non-Shen Embroidery images. The resulting confusion matrix was shown in Fig. 9, where (a) represented AlexNet, (b) represented VGG16, (c) represented MobileNet V1, (d) represented InceptionV3, and (e) represented ResNet50.From the confusion matrix, it can be observed that the MobileNet V1 model correctly recognized 98 images and misclassified 2 images of Shen Embroidery. The experimental results indicate that all five models can accurately recognize the Shen Embroidery. However, MobileNet V1 was more accurate in distinguishing between "shenxiu" and non-"shenxiu" images. Therefore, the MobileNet V1 model was selected as the base model for the experiments in this study.

### Comparison of the transfer learning before and after

**Figure 10:**
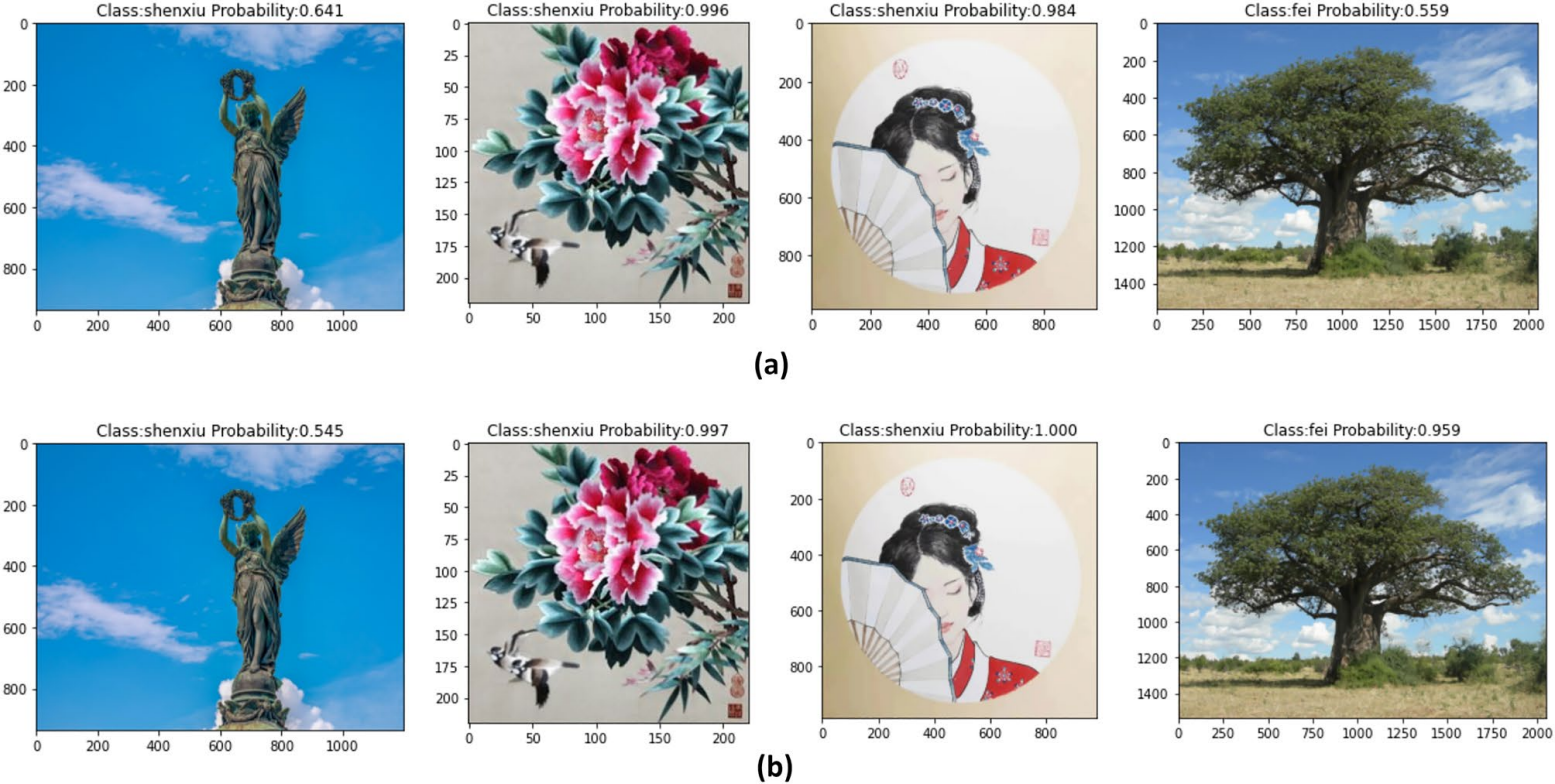
Recognition effects of the transfer learning before and after.

Due to the limited number of artworks in Shen Embroidery, along with an insufficient image dataset, the recognition rate is low. This study introduced transfer learning. In the training process, the pre-trained model of MobileNet V1 was loaded. The dataset, parameters, and experimental equipment were kept unchanged during the training. The experimental results of the transfer learning before and after were shown in Fig. 10. Where, (A) represented the results without transfer learning and (B) represented the results with transfer learning. From the recognition result images, it can be observed that both models, transfer learning before and after, made an error in recognizing the first image by misclassifying the "fei" image as a "shenxiu" image. However, compared to the recognition result for the fourth image, the confidence level of identifying the "fei" image was 55.9% in (A) and 95.9% in (B). This indicated that the model after transfer learning can more accurately recognize "fei" images. The experimental results of the transfer learning before and after were shown in Table 3. The average recognition accuracy of the MobileNet V1 (transfer learning) model was 97.86%, which was 1.11% higher than the MobileNet V1 model. The experimental data shown that the recognition model with transfer learning can more accurately identify "shenxiu" images.

**Table 3:** Experimental results of the transfer learning before and after.

Model	Accuracy (%)
MobileNet V1	96.15
MobileNet V1(transfer learning)	97.26

### Recognition results of the improved MobileNet V1

**Figure 11:**
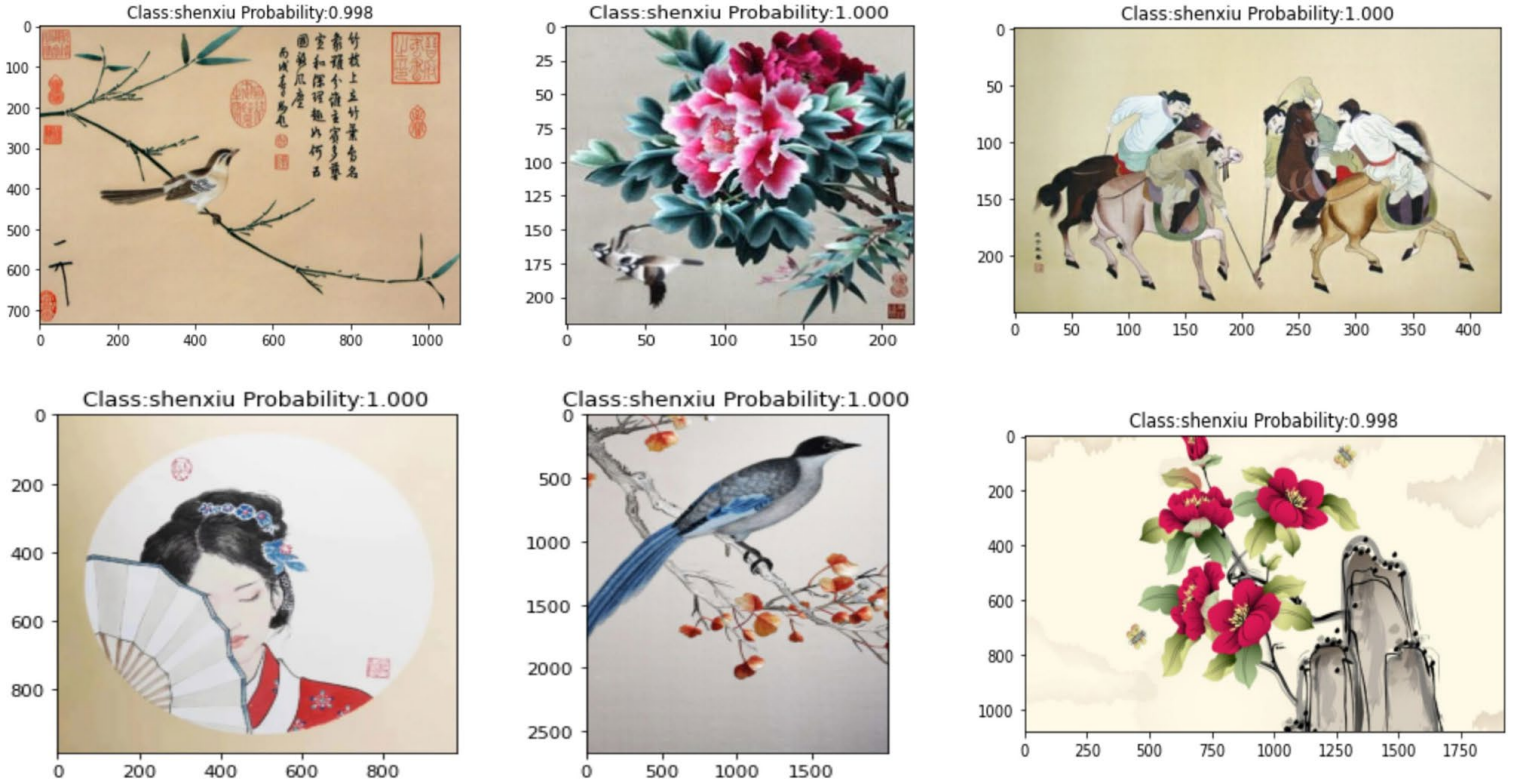
The recognition results of the Shen embroidery.

**Figure 12:**
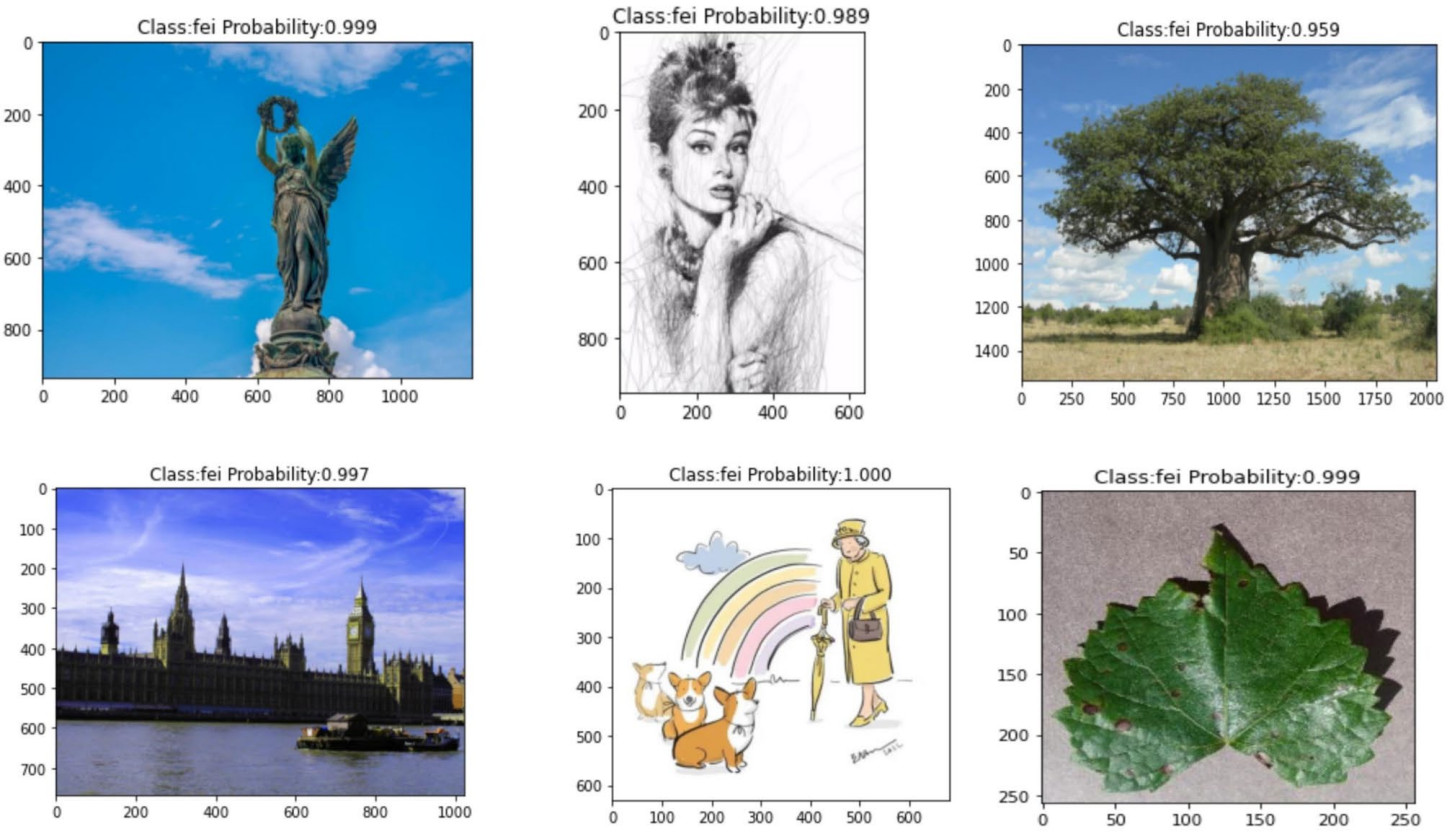
The recognition results of the non-Shen embroidery.

The MobileNet V1 model has been improved in this study to enhance the recognition accuracy of Shen Embroidery. The same dataset was used to train the Improved MobileNet V1 model through transfer learning, resulting in a recognition accuracy of 98.45% after model fitting. To validate the accuracy of the fitted model, a test dataset containing 100 images with Shen Embroidery was used. The recognition results of the improved MobileNet V1 for Shen Embroidery and non-Shen Embroidery were shown in Figs.11 and 12. The Fig. 11 represented the recognition results for Shen Embroidery, while the Fig. 12 represented the recognition results for non-Shen Embroidery. From the recognition results in Fig. 11, it can be observed that the improved MobileNet V1 model can almost perfectly identify Shen embroidery images. Compared to the MobileNet V1 model, the improved MobileNet V1 model also exhibits a higher confidence level in correctly identifying Shen embroidery images. As shown in Fig. 12, the improved MobileNet V1 model can also identify non-Shen embroidery images. In Fig. 12, various types and categories of daily life images are correctly identified as "fei" by the improved MobileNet V1 model. Similar to Shen embroidery images, the improved MobileNet V1 model demonstrates higher confidence in correctly identifying non-Shen embroidery images compared to the MobileNet V1 model. Through comprehensive comparison, experiments confirm that for innovative applications targeting Shen embroidery images, the improved MobileNet V1 model proposed in this paper, based on convolutional neural networks, can effectively identify Shen embroidery images.

## Conclusion

This study proposed an improved MobileNet V1 based on CNN for the recognition of Shen Embroidery in Nantong, a non-material cultural heritage. In this paper, five image classification models were experimented and compared. Ultimately, the MobileNet V1 model was selected as the recognition network for Shen Embroidery. Furthermore, the experimental dataset was enhanced to address the challenges of limited Shen Embroidery works and low recognition rates. In the experimentation process, transfer learning was applied to the MobileNet V1 network to accelerate the model fitting during training. Finally, the avg pool in the MobileNet V1 network was replaced with SPP to better extract features from Shen Embroidery.The experimental results demonstrated that the improved MobileNet V1 achieved a recognition accuracy of 98.45%, which was 2.3% higher than the original network. This validated that the improved MobileNet V1 can accurately identify Shen Embroidery. Innovative research on Shen Embroidery contributes to the application of artificial intelligence technology in the protection and inheritance of non-material cultural heritage.

## Data Availability

The datasets generated during and/or analyzed during the current study are available from the corresponding author on reasonable request. The partial dataset is available on https://www.scidb.cn/en/s/JR7ZJn.
